# Leader’s Implicit Followership and Employees’ Innovative Behavior: Chain Mediation Effect of Leader–Member Exchange and Psychological Empowerment

**DOI:** 10.3389/fpsyg.2022.815147

**Published:** 2022-05-19

**Authors:** Wei Liang, Chen Lv, Yongchang Yu, Tingyi Li, Peng Liu

**Affiliations:** ^1^School of Tourism, Taishan University, Taian, China; ^2^Business Administration College, Shandong Technology and Business University, Yantai, China

**Keywords:** leader’s implicit followership, employees’ innovative behavior, leader–member exchange, psychological empowerment, chain mediation effect

## Abstract

In the Chinese society, where power distance is high, leaders’ attitudes and behavior toward employees determine their career development as well as affect the entire team’s performance. Therefore, exploring the kind of employees that leaders expect in China is essential. Based on implicit followership theory perspective, this study considers leaders’ positive implicit followership (LPIF) as the main research variable and examines its influence on employees’ innovative behavior (EIB). Moreover, it explores the multiple mediation effect of the leader–member exchange (LMX) relationship and psychological empowerment (PE) in this influence mechanism. The study sample comprised 389 leaders and their direct employees at 45 large- and medium-sized enterprises in Shandong, Beijing, Hebei, Shanghai, Shanxi, Zhejiang, and other regions of China. We used the leader–employee 1:1 matching questionnaire, and the longitudinal research design was adopted to avoid homology variance, making the study results more realistic and reliable. This study used the SPSS 26.0 and AMOS 26.0 statistical software to verify the hypotheses. Our findings show that LPIF has a significant positive effect on EIB, and LMX and PE have multiple mediation effects on the relationship between LPIF and EIB. When the level of LPIF is high, LMX and PE are also enhanced, which in turn promotes the increase in EIB. This study provides a new perspective for subsequent research on the psychological mechanism of employees and suggests an important method for understanding leadership and following processes in an organization. It plays a guiding role for the management practice of an enterprise, selection of leaders, and training of employees.

## Introduction

As per Hofstede’s (2001) cultural dimension theory, the power distance index in Chinese society is much higher compared to Western countries. In China, people’s acceptance of power inequality is also higher. Power distance refers to the degree to which members of an organization accept the uneven distribution of power. In Chinese society, where power distance is high, leaders’ attitudes and behavior toward employees determines their career development as well as affects the entire team’s performance (Ni et al., 2019). Therefore, it is essential to explore the kind of employees that leaders expect in China. Leaders’ implicit followership refer to their perceptions of employees’ qualities and behaviors (Sy, 2010). Furthermore, it is a leader’s cognitive schema for employees, representing employees’ characteristics in leaders’ minds (Sy, 2010). Leaders’ implicit followership includes positive implicit followership (prototype, LPIF) and negative implicit followership (anti-prototype, LNIF; Van Gils et al., 2010). LPIF is a leader’s positive assumption about the characteristics and behaviors that employees should have (Whiteley et al., 2012; Epitropaki et al., 2013). In China, the measurement of implicit followership focuses on LPIF research. This is because with the continuous development of enterprises, employees who conform to LNIF will be eliminated by the enterprise (Zhu et al., 2017). Simultaneously, research on LPIF is also consistent with the mainstream direction of research (Kong et al., 2018) and can play a positive role in management practice (Duong, 2011). Therefore, this study also focuses on leaders who have positive expectations from their employees.

According to implicit followership theories (IFTs), leaders have preconceived notions of employees and make judgments based on this perception to influence others (Fiske and Taylor, 1991). Leaders internalize and endorse a certain implicit followership and gradually use this fixed standard to select, evaluate, and treat employees (Shondrick and Lord, 2010), while employees tend to act according to their leaders’ expectations (Eden, 1992). Leaders’ expectations or perceptions affect their attitudes or behaviors toward employees and ultimately influence their behavior (Lord and Maher, 1990; Rosenthal, 1993). Eventually, different levels of LPIF bring about differences in employee behavior (Carsten et al., 2010).

Scholars have investigated the relationship between LPIF and leaders’ perception and behavior as well as employees’ work attitude and behavior from multiple perspectives. For example, studies have found that LPIF can promote leaders’ trust and liking for employees (Sy, 2010) and can improve the level of leader–member exchange (LMX; Uhl-Bien and Pillai, 2007). Moreover, LPIF has a positive relationship with employees’ trust and liking for leaders, employees’ job performance, and team creativity (Sy, 2010; Whiteley et al., 2012; Kong and Qian, 2015; Zhu et al., 2017, 2019).

Employees’ innovative behavior (EIB) is vital to ensure the survival and success of enterprises and is an important capital of individuals and enterprises (Kanter, 1983; West and Farr, 1990). EIB is the premise and foundation of enterprise innovation (Woodman et al., 1993; Shalley et al., 2004); it can also improve organizational performance (Dedahanov et al., 2017). However, the existing research on the improvement of employee creativity has mostly focused on antecedent variables, such as external conditions of the enterprise (Dul and Ceylan, 2011) and explicit leadership theory (Jaiswal and Dhar, 2016), and research on EIB from an implicit perspective is scant. Shalley et al. (2004) and Zhang and Bartol (2010) believe that one responsibility of a leader is promoting the formation of EIB and ultimately obtaining sustainable competitive advantage and achieve organizational success. To grasp the mechanism of EIB, it is important to identify the subjective and objective factors that affect EIB within the organization and introduce new concepts or theories. Studying EIB from the LPIF perspective is conducive for maintaining the consistency and coherence of innovative ideas and innovation activities between senior managers and employees so that enterprises have sustainable competitive advantages.

This study attempts to verify how LPIF affects EIB and explores the chain mediation role of LMX and psychological empowerment (PE) in this influencing mechanism.

## Theoretical Background and Research Hypotheses

### Leaders’ Positive Implicit Followership and Employees’ Innovative Behavior

Sy (2010) defines positive implicit followership as employees’ expectations or assumptions about positive characteristics or behaviors. Essentially, it is a positive expectation of employees (Whiteley et al., 2012). Thus far, the subvariable of positive implicit followership adopted by most scholars is Sy’s (2010) three-dimensional classification method, that is, “Industry, Enthusiasm, and Good Citizen.” Specifically, in terms of working ability, an employee is hard-working, honest, and outstanding. Emotionally, they work with enthusiasm, positivity, and fun. In terms of interpersonal relationships, employees get along well, exhibit good teamwork, and share a sense of trust (Sy, 2010; Peng et al., 2016). In this study, the aforementioned three-dimensional classification methods are described as “ability,” “emotion,” and “interpersonal relationship.” In “ability,” individuals can enhance their ability to achieve high-performance expectations. Amabile (2012) indicated that the stronger the individual’s ability in associated fields, the more innovative thinking and actions can be triggered. In “emotion,” leaders’ expectation of employees’ positive emotion encourages them to express themselves actively in the organization (Kruse and Sy, 2011) or evoke high self-efficacy (Wang and Wang, 2015). Therefore, these positive and affirmative emotions can promote employees’ forward-thinking when individuals face the pressure of failure brought about by innovation, which positively impacts innovation behavior (Frijda, 1986). In “interpersonal relationship,” the positive expectation of the employee’s “interpersonal relationship” can enable them to manage interpersonal relationships more carefully in the organization, thereby reducing conflict and improving the quality of the interpersonal relationship. Good interpersonal relationships can form an excellent psychological atmosphere for employees (Scott and Bruce, 1994), help information sharing between individuals and groups, and accelerate the construction of personal knowledge systems (Hakanen et al., 2008). Furthermore, such relationships can promote innovative behavior and improve learning ability (Andrews and Delahaye, 2000).

Sy’s (2010) empirical study points out that leaders with positive views on employees have positive role expectations. A leader’s role expectation and innovation support are essential factors for employees to implement innovation behavior (Scott and Bruce, 1994). When employees have low role expectations, they tend to complete tasks and not show exploratory behaviors, such as thinking about new ideas. Conversely, employees with positive role expectations are more likely to show exploratory behaviors, such as thinking about new ideas (Derler and Weibler, 2014). Employees’ out-of-character behavior is consistent with leaders’ expectations of employees’ roles, which triggers and activates positive concepts, such as being referred to as a “good employees” and an “insiders” (Bargh et al., 1996). Chinese scholars Wang and Li (2017) and Zhu et al. (2017) have verified the influencing relationship between LPIF and EIB. Based on this, the following hypothesis is proposed:

H1: Leaders’ positive implicit followership has a significantly positive effect on EIB.

### The Mediation Effect of Leader–Member Exchange

Specifically, social exchange theory (Blau, 1964) describes the social exchange of tangibles (i.e., money) and intangibles (i.e., social support) as a social exchange process. This theory has been applied to illuminate numerous circumstances and behaviors. For example, it is used to explain employees’ job performance (Kuruzovich et al., 2021), interorganizational exchanges and trust (Lioukas and Reuer, 2015), and the relationship between leader and peers (Miao et al., 2014). Moreover, social exchange theory provides a basis for understanding the relationship between leaders and employees and explains the influence of LMX on employees’ attitudes and behaviors. With limited time and energy, leaders can communicate with each employee at different levels at work. Some employees gain the leader’s trust to become insiders, while others become outsiders (Graen and Uhl-Bien, 1995). Additionally, leaders who have positive assumptions about their employees (LPIF) can give them more trust, support, and encouragement (Sy, 2010). The deeper the trust, the higher the LMX level (Kong and Qian, 2015). Thus, the leader will regard the employees consistent with LPIF as insiders (Duong, 2011; Whiteley et al., 2012). For employees consistent with LPIF, leaders will recognize their out-of-role behaviors, thus enabling leaders and employees to develop closer relationships and trust each other more. Leaders can provide more information and resources to “insiders” (Scandura and Schriesheim, 1994), which are not only crucial for the inception of creativity (Lin et al., 2018) but also positively impact EIB (Scott and Bruce, 1994; Basu and Green, 1997; Tierney et al., 1999). In high-quality exchange relationships, the leader shares more constructive and comprehensive ideas with the employees (He et al., 2021). Meanwhile, employees who experience high-quality exchange relationships with their leader are more motivated and more likely to enjoy autonomy while dealing with challenging tasks (Lin et al., 2018; Kirrane et al., 2019). Risk-taking in new procedures and experimenting with novel ideas lead to superior creativity for employees. A high-level LMX can stimulate employees’ positive work response and improve enthusiasm for EIB (Atwater and Carmeli, 2009), thus helping employees to innovate (Graen and Wakabayashi, 1994). Based on this, the following hypothesis is proposed.

H2: Leader–member exchange plays a mediation role in the relationship between LPIF and EIB.

### The Mediation Effect of Psychological Empowerment

Psychological empowerment is a process that can increase intrinsic motivation and enable employees to control their lives (Spreitzer, 1995); it is an individual’s perception regarding job meaning, self-efficacy, self-determination, and job impact (Thomas and Velthouse, 1990). Individuals are motivated only if the task itself brings a sense of self-determination and competence to the individual (Deci, 1975). PE theory states that employees’ perceptions concerning work will affect their behavior (Spreitzer, 1995). Leaders with LPIF exhibit more positive attitudes and behaviors and give their employees more trust, attention, and empowerment (Junker and van Dick, 2014; Peng and Wang, 2015; Yang and Peng, 2015). This makes employees feel greater self-confidence and work meaning; consequently, as employees have the ability and belief of self-determination, they trust themselves to determine the process and results of own work behavior (Kong and Qian, 2015). Employees with high levels of PE have greater autonomy and influence over their work, feel less restricted than other employees, and tend to be more proactive and innovative (Amabile, 1988). PE can trigger EIB by improving self-efficacy, strengthening intrinsic motivation, and increasing autonomy (Laschinger and Shamian, 1994). Therefore, from the self-determination perspective, employees’ basic autonomy needs in terms of autonomy, competence, and relatedness are satisfied; therefore, psychologically empowered employees obtain a higher level of intrinsic motivation (Ryan and Deci, 2000) and thus are more likely to propose new ideas and implement incremental innovation (Singh and Sarkar, 2012). Additionally, evidence shows that self-determined and impactful employees are more likely to test new ideas (Schermuly et al., 2013). Moreover, employees who believe in their competence are more creative (Zhou, 1998) and those with meaningful commitment to their tasks exhibit innovative behavior (Bass, 1985; Singh and Sarkar, 2012).

H3: Psychological empowerment plays a mediation role in the relationship between LPIF and EIB.

### The Mediation Chain Effect of Leader–Member Exchange

As per self-determination theory (Deci and Ryan, 1985), employees will judge the quality of exchanges with leaders based on their own perceptions (Kong and Qian, 2015). When the LMX relationship level is high, employees become the focus of the leader’s attention and gain a positive influence (Dansereau et al., 1995), and they perceive themselves as having high autonomy (Liden and Graen, 1980). High LMX indicates mutual respect, liking between the parties, and positive interaction with followers, which extend beyond the formal job description (Nahrgang et al., 2009). Conversely, subordinates who perform only in accordance with the prescribed employment contract are characterized as “out-group,” with limited reciprocal trust and support and few rewards from their supervisors (Deluga, 1998). Employees with low LMX encounter a diminished scope for PE (Aggarwal et al., 2020). Previous research has shown that the quality of the relationship between leaders and employees influences employees’ perceived levels of PE (Harris et al., 2009; Hill et al., 2014). A high level of LMX helps improve the PE of employees (Aggarwal et al., 2020), and PE will further lead to various organizational consequences, such as EIB (Hill et al., 2014; Wang et al., 2016; Newman et al., 2017; Hu et al., 2018). Thus, the following hypothesis is proposed.

H4: Leader–member exchange and PE play a mediation chain role in the relationship between LPIF and EIB.

## Research Design and Methods

### Research Model

**FIGURE 1:**
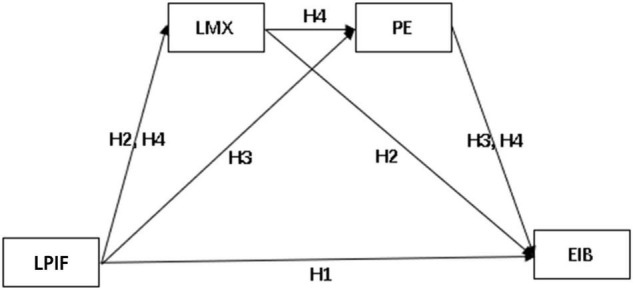
Theoretical model. Note: LPIF, leaders’ positive implicit followership; LMX, leader–member ex-change; PE, psychological empowerment; and EIB, employees’ innovation behavior.

An analysis of the existing literature, combined with research hypotheses, Build LPIF, LMX, PE, and EIB influence mechanism model (Figure 1).

### Operational Definition and Measurement of Variables

A classical scale with high international reliability is used to measure variables in order to ensure the validity of the measurement. The scale used in this study has been widely used in the Chinese context and has high reliability and validity. A 5-point Likert scale was used for measurement, ranging from “completely inconsistent” to “completely consistent.” Operational definitions and measurement scales of the main variables are as follows.

1. Leaders’ positive implicit followership. Leaders were asked to rate what they expect from their employees by using a nine-item questionnaire developed by Sy (2010). The sample items consist of “industry, enthusiasm,” etc.
2. Employees’ innovative behavior. This study uses the EIB questionnaire for nine items developed by Janssen (2000). For example, “I often introduce new ideas into the work environment.” Employees self-evaluate this scale.
3. Leader–member exchange. This study uses the LMX questionnaire for seven items developed by Graen and Uhl-Bien (1995). For example, “I get along well with my boss and can work efficiently together.” Employees self-evaluate this scale.
4. Psychological empowerment. The PE perceptions of employees were measured through the 12-item scale developed by Spreitzer (1995). For example, “What I have done is very meaningful to me.” Employees self-evaluate this scale.
5. To improve the accuracy of the analysis results, control variables include eight projects: leaders’ gender, age, education level; employees’ gender, age, education level, and income; time spent together by leaders and employees.

### Sample Characteristics

In this study, the survey participants are 450 leaders and their corresponding employees from 45 large- and medium-sized enterprises in Shandong, Beijing, Hebei, Shanghai, Shanxi, Zhejiang, and other places. Before distributing the questionnaire, we contacted 45 managers or manager-level supervisors from the target company *via* telephone or the internet. These leaders examined their corresponding employees within the organization who could participate in this research. The ratio of leaders to employees is 1:1. The most significant feature of this study is that data were collected in two time waves through a time lag approach. At Time 1, we distributed LPIF questionnaires to leaders, and at Time 2 (i.e., 3 months later), we distributed EIB, LMX, and PE questionnaires to employees. We chose a 3-month interval when investigating the influence of LPIF to fully observe the impact of LPIF on outcome variables while reducing CMV (Podsakoff et al., 2003).

Of the 450 matched questionnaires distributed, 406 were collected after excluding invalid questionnaires. In total, 389 valid questionnaires for leader–employee matching were obtained, with a valid response rate of 86.4%. Regarding sample size adequacy, Bentler and Chou (1987) suggested that the appropriate sample size is about 5–10 times the number of items to perform structural equation modeling (SEM). The sample size used in this study (*N* = 389) was much larger than those calculated by the SEM software, and it fulfilled standards recommended by previous scholars (Bentler and Chou, 1987; Kock and Hadaya, 2016; Kyriazos, 2018). Therefore, the sample size of this study seemed adequate and justified to perform SEM for data analysis, fulfilling the minimum sample size requirement.

In the leadership sample, the proportion of male leaders was 70.4%, much higher than that of female leaders (29.6%). The age composition was 40–50 years (43.2%) and 30–40 years (28.5%). In terms of education level, leaders with a bachelor’s degree accounted for the most significant proportion (58.6%). The largest proportion of time spent working together for leaders and employees was 1–3 years (37.6%).

In the employee sample, men accounted for a larger proportion (58.6%). Regarding age, the proportions of 20–30-year-olds (40.9%) and 30–40-year-olds (36.2%) were the highest, totaling 77.1%. A bachelor’s degree accounted for the highest proportion (58.4%) in terms of educational level. Regarding employee monthly salary, 54.2% of employees had a monthly salary of between 5,000 and 10,000 RMB.

### Analysis Method

This study used SPSS 26.0 and AMOS 26.0 for empirical analysis. The structure model constructed by AMOS 26.0 was mainly used for confirmatory factor analysis of variables, analysis of convergence validity, and discriminative validity of measurement models.

SPSS 26.0 performs descriptive statistics, reliability analysis, correlation analysis between variables, and regression analysis to verify main effects. This study used SPSS’s plug-in process macro program to analyze the intermediary and chain intermediary effects of the LMX relationship and PE.

## Empirical Analysis

### Common Method Variance

We collected data from the respondents through the self-reported method; therefore, an issue of common method bias may exist (Podsakoff et al., 2003). To reduce common method variance, the current study adopted two methods. First, for questionnaires, the scale was paginated, and an appropriate rest time was provided between answering questions on each page. Thus, the resulting time difference reduced the influence of common method variance caused by the same continuity scale (Podsakoff et al., 2003; Aggarwal et al., 2020). Second, data were collected in two time waves through a time lag approach, with only the independent variable (LPIF) measured at Time 1. At Time 2, after a 3-month interval, mediator variables (LMX and PE) and dependent variables (EIB) were measured. Collecting data in this manner may reduce the impact of CMB (Atwater and Carmeli, 2009).

Then, we used two methods to check for CMV. First, we used Harman’s single factor test for the common method bias test. The result of unrotated factor analysis shows that four factors with feature roots greater than 1 were extracted. The variance explanation rate of the first common factor is 33.26%, which is less than 40% proposed by Podsakoff et al. (2003). The judgment standard indicates that there is no apparent common method bias in this study’s data. Second, we completed the correlation coefficient test of latent variables (Table 2). The absolute value of the correlation coefficient between latent variables is ≤0.672, far less than 0.9, indicating no significant common variance deviation in the research data (Podsakoff et al., 2003; Yang et al., 2022). The analysis indicates that CMV does not pose any risk or concerns for the results of this study.

### Validity Test

To verify the validity and reliability of the questionnaire used, SPSS26.0 was used to conduct factor and reliability analyses. The measurement results show that the reliability of LPIF, EIB, LMX, and PE scales are 0.778, 0.825, 0.873, and 0.919, respectively, and the Kaiser-Meyer-Olkin values of each scale are 0.758, 0.790, 0.886, and 0.92, respectively. Bartlett’s test is significant (*P* < 0.000), and the reliability and validity are above 0.7. All scales show good reliability and validity. Each question item corresponds to each corresponding factor, indicating the scale has high construct validity.

We use Amos26.0 to verify the convergent validity of variables. First, the factor loads of the four variables in the model corresponding to each topic are greater than 0.5, and the Average variance extracted (AVE) value of each variable is between 0.504 and 0.564. The Construct Reliability (CR) value is between 0.753 and 0.939 (Table 2), indicating good convergent validity. Second, we performed confirmatory factor analysis (Table 1). Compared with other factor models, the four-factor model has the best goodness of fit. Root mean square error of approximation is less than 0.05, *X*^2^/df is less than 3, and Goodness-of-fit index and Comparative-fit index are greater than 0.8. All values are within the required range, indicating that the structural validity between variables is also at a good level.

**TABLE 1:** Confirmatory factor analysis results.

Model	*x*^2^/df	RMR	GFI	RMSEA	CFI	PGFI
Four-factor model (LPIF, LMX, PE, EIB)	1.856	0.053	0.897	0.047	0.803	0.742
Three-factor model (LPIF + LMX, PE, EIB)	2.147	0.095	0.880	0.054	0.662	0.736
Three-factor model (LPIF + PE, LMX, EIB)	3.188	0.052	0.843	0.075	0.865	0.706
Two-factor model (LPIF + LMX + PE, EIB)	6.323	0.089	0.633	0.117	0.670	0.534
Single factor model (LPIF + LMX + PE + EIB)	6.304	0.089	0.632	0.117	0.669	0.535

*LPIF, leaders’ positive implicit followership; LMX, leader–member exchange; PE, psychological empowerment; and EIB, employees’ innovation behavior.*

**TABLE 2:** Correlation analysis of variables and discriminant validity analysis.

	Mean	SE	1	2	3	4	AVE	CR
1. LPIF	3.50	0.63					0.504	0.753
2. LMX	3.51	0.67	0.474**				0.543	0.892
3. PE	3.53	0.71	0.498**	0.370**			0.541	0.939
4. EIB	3.57	0.65	0.672**	0.533**	0.587**		0.564	0.912

LPIF, leaders’ positive implicit followership; LMX, leader–member exchange; PE, psychological empowerment; and EIB, employees’ innovation behavior. **P < 0.01.

### Correlation Analysis

In Table 2, the correlation coefficient *r* value between the variables is mostly between 0.3 and 0.6, and the significance level is below 0.05. The highest correlation coefficient between the variables in this study is 0.672, which is lower than 0.7, indicating no multicollinearity among the variables. Next, we performed discriminant validity analysis. The square of the highest (LPIF and EIB) correlation coefficient between the variables is 0.452, lower than the lowest value of 0.504 (LPIF’s AVE value) in the AVE values, verifying the discriminant validity of this research model. In this study, the correlation between the latent variables is significant, and subsequent empirical analysis can be conducted.

### Hypothesis Testing

#### Testing of the Main Effect

The *F* value in Table 3 is significant, indicating that the variable is suitable for regression analysis. The standardized regression coefficient of LPIF and EIB is β = 0.658, and the significance is *P* < 0.001. Therefore, LPIF has a significant positive effect on EIB, thus supporting Hypothesis 1. The Variance Inflation Factor values of Models 1 and 2 are less than 3. The Durbin-Watson value is significant at the 0.05 level, indicating that the model analysis results are acceptable.

**TABLE 3:** Main effects test.

		EIB	
		M1	M2
Leaders’	Gender	0.149**	0.015
	Age	0.153**	0.004
	Education level	0.205***	0.038
Employees’	Gender	0.007	0.044
	Age	0.038	–0.023
	Education level	0.207***	0.151***
	Income	0.053	0.047
	Working time with leaders	–0.087	–0.051
LPIF			0.658***
*R* ^2^		0.145	0.487
Adjusted *R*^2^		0.127	0.475
*F*		8.050***	39.963***

LPIF, leaders’ positive implicit followership; and EIB(, employees’ innovation behavior. **P < 0.01; ***P < 0.001.

#### Testing of the Mediation Effect

Following Hayes et al. (2011), Hayes (2013), and Chen et al. (2013), we used the SPSS plug-in process macro program to test whether the mediation effect of LMX and PE between LPIF and EIB are significant as well as examine the chain mediation effect of the LMX and PE. This method has been verified in many studies (Liu et al., 2019; Shang et al., 2021; Yang et al., 2022). We set bootstrap sampling at 5,000 and chose bias correction to calculate total, total direct, and total effects. If the 95% CI of the standardized path coefficient does not contain 0, it indicates a significant mediation effect. The results are shown in Table 4. The total effect is 0.610, at a 95% significance level; the bootstrap confidence interval is [0.535, 0.686], and the total direct effect value is 0.370. At a 95% significance level, the bootstrap confidence interval is [0.293, 0.448]. The total indirect effect value is 0.240, at a 95% significance level, and the bootstrap confidence interval is [0.185, 0.300]. None of the confidence intervals of the aforementioned effect values contain 0, indicating that the overall mediation effect is significant.

**TABLE 4:** Bootstrap analysis of significance test of mediation effect.

Type	Effect value	Relative effect value	Bootstrap SE	Bootstrap CI	
				Lower	Upper
Total effect	0.610		0.038	0.535	0.686
Direct effect	0.370		0.039	0.293	0.448
Indirect effect (Total)	0.240		0.029	0.185	0.300
1.LPIF→LMX→EIB	0.115	47.9%	0.022	0.075	0.159
2.LPIF→PE→EIB	0.100	41.7%	0.018	0.068	0.139
3.LPIF→LMX→PE→EIB	0.024	10.0%	0.007	0.011	0.041

*LPIF, leaders’ positive implicit followership; LMX, leader–member exchange; PE, psychological empowerment; and EIB, employees’ innovation behavior.*

Specifically, on the “LPIF→LMX→EIB” mediation path, at a 95% significance level, the indirect effect value is 0.115. The confidence interval is [0.075, 0.159], excluding 0. This indicates that the mediation effect is significant, thus supporting Hypothesis 2.

On the “LPIF→PE→EIB” mediation path, at a 95% significance level, the indirect effect value is 0.100. The confidence interval is [0.068, 0.139], excluding 0, indicating that the mediation effect is significant, thus supporting Hypothesis 3.

For the chain mediation path, “LPIF→LMX→PE→ EIB,” at a 95% significance level, the indirect effect value is 0.024. The confidence interval is [0.011, 0.041], excluding 0, indicating LMX and PE play a chain mediation role in the relationship between LPIF and EIB. As such, research Hypothesis 4 is supported.

## Summary, Implications, and Discussion

### Summary

The study participants included 389 leaders and their matching employees from 45 large- and medium-sized enterprises in China. Through empirical analysis, we verified the influencing mechanism of leaders’ implicit following and EIB, summarized as follows.

First, LPIF has a positive (+) effect on EIB. When a leader has high positive expectations for the characteristics of employees, it will promote the generation of EIB, and it is difficult to promote EIB when leaders have low LPIF of employees. This finding is consistent with that of Kong and Qian (2015).

Second, the LMX relationship plays a mediation role between LPIF and EIB. Leaders’ positive expectations for employee characteristics will lead to more care and trust in employees (Sy, 2010). Leaders regard employees who meet their expectations as “insiders” who are willing to establish positive emotional connections with their leaders (Duong, 2011; Whiteley et al., 2012). Such insiders will be willing to take more out-of-role behaviors to repay the leader’s care and trust (Hoption et al., 2015). When employees feel a better exchange relationship with the leader, they will improve their innovative behavior to promote corporate innovation. Conversely, leaders view employees who are dissatisfied with their LPIF as outsiders, because their leaders give fewer benefits to outsiders, communicate less, and trust outsiders less (Schneider, 1987). This is not conducive to the generation of EIB.

Third, PE plays a mediation role between LPIF and EIB. Leaders’ positive expectations can improve employees’ PE, thereby enhancing EIB. In other words, the process of implicit followership will affect employees’ PE, which in turn affects employees’ interpretation of LPIF. This causes employees to have different feelings about leadership, thus leading to varying outcomes. Leaders with high LPIF will give employees more care, love, trust, and empowerment (Sy, 2010), thus making employees feel greater self-determination, self-confidence, work meaning, and self-efficacy, which in turn promotes the generation of employee EIBs (Kong and Qian, 2015).

Fourth, LMX and PE play a chain mediation role in the relationship between LPIF and EIB. Leaders’ positive expectations of employee characteristics promote high-quality exchange relationships between leaders and employees. Meanwhile, employees with high LMX perform better in the organization, have a stronger perception of the work environment and a positive attitude to accept work challenges, and demonstrate innovative spirit (Aggarwal et al., 2020). When employees feel highly empowered in terms of meaning in the workplace, they feel more confident in their abilities and strive to achieve high levels of self-actualization (Gerstner and Day, 1997; Gomez and Rosen, 2001). In a high-quality LMX relationship, there is generally a sense of mutual trust and respect between the leader and members (Aggarwal et al., 2020). In return, leaders enhance their empowering working conditions, such as providing scarce resources and flexibility in decision-making. Previous research suggests that employees with a good relationship with their leaders perform better than those with a poor relationship; employees also have a strong ability to adapt to change (Liden et al., 2000; Chen and Klimoski, 2003; Carson and King, 2005).

### Implications

First, this study enriches IFTs. LPIF provides a more in-depth analysis of the leadership process regarding how leaders and employees perceive, decide, and act. This concept broadens the application of IFTs in management.

Second, this study leads through the research on LPIF in EIB and reveals the internal connection between the two factors. Furthermore, it focuses on the role of LMX and employee PE in this process, providing a clue that human resource management should pay attention to employees’ feelings toward leadership.

Research on the mediation effect of LMX shows that in the context of Chinese enterprise management, employees value exchange with leaders in social exchange relationships: whether the relationship with leaders is good is directly related to employees’ attitudes and behaviors, which affects employees’ psychological feelings and career development. Further, performance differences among employees stem largely from leaders’ perceptions of employees and subsequent interactions (Collinson, 2006); thus, there is a need to strengthen LPIF research.

This study has particular significance to business management practice. First, because LPIF and EIB are significantly positively correlated, employee innovation is essential for organizations to obtain sustainable competitive advantages in a dynamic and changeable market environment. To further promote EIB, organizations can preferentially select leaders who have positive expectations of employees, that is high implicit followership. When developing leaders in lower-level business organizations, stakeholders must focus on developing their knowledge of employees’ positive perceptions.

Second, based on social exchange theory, LPIF influences the behavior of employees through social exchange relationships (Kong and Qian, 2015). The relationship between employees and their leader plays a vital role in the leader’s perception of employees and their behaviors. The current study findings can help managers reduce negative emotions among employees and enhance positive emotions related to work and organization. As the spokesperson of the organization, the leader should pay attention to the contribution and happiness of the employees in influencing behavior and provide opportunities for employees to participate in the communication process with the leader, formulate policies, and make contributions. Such actions will improve employee performance by establishing high-level exchange relationships.

Third, employees who conform to LPIF are “insiders” who leaders like. For employees, after becoming “insiders,” they will engage in work that is valuable to the achievement of organizational goals and enhance their sense of belonging and identity (Eisenberger et al., 1990). Moreover, they will thus have better performance at work (Eisenberger et al., 1986). The role of LPIF in Chinese enterprise management is affected by employee PE level; hence, organizations should emphasize how employees feel about their leaders.

## Discussion

Although the current study provides valuable information pertaining to the variables under consideration, there are still some limitations, which need to be considered while generalizing the study results. (1) All variables in this study were obtained through self-assessment methods; therefore, there might be an issue of CMB. To handle this limitation, we collected the data in two phases. At the first point, we collected data for the independent variables, and at the second point, we collected the data for the mediator and dependent variables. Besides this, CMB is not a major concern in studies that use well-designed multifactor statements (Spector, 1987). Although researchers have tried their best to minimize the effect of CMB, each remedy has its own disadvantages (Podsakoff et al., 2003). To better test the proposed hypotheses and improve the reliability of the study results, follow-up research can consider a combination of self-evaluation and other evaluation methods. (2) Future studies needs a more comprehensive perspective; specifically, we should focus on investigating leadership, employees, context, hierarchy, and their dynamic interactions. Future studies can examine the impact of LPIF on employee behavior from the perspective of implicit followership matching and cognitive differences. (3) It is necessary to study different groups of people—for example, knowledge-based employees, new generation employees, etc.—to explore how different types of employees form their followers, how to affect their interaction with leaders, and how they achieve their performance. Future research will be crucial to promote the development of leadership research.

Based on the foundation of previous studies, this study builds a chain mediation model, focusing on how LPIF affects EIB. It offers a new perspective for scholars to examine the psychological mechanisms of EIB. Further, it provides an important method for understanding the process of leadership and followers in organizations. Additionally, this study has a certain guiding effect on employee selection and training. A crucial innovation of this study is that it ensured the rigor of the research design by adopting the following approaches: (1) the leader–employee 1:1 matching questionnaire and (2) longitudinal research design, which helped avoid common method deviations as much as possible. Therefore, the present study’s results are accurate and reliable, which can be further generalized to a large extent.

## Data Availability Statement

The raw data supporting the conclusions of this article will be made available by the authors, without undue reservation.

## Author Contributions

WL: conceptualization, methodology, writing—original draft. CL: writing—review and editing. YY: resources. TL and PL: software. All authors approved it for publication.

## Conflict of Interest

The authors declare that the research was conducted in the absence of any commercial or financial relationships that could be construed as a potential conflict of interest.

## Publisher’s Note

All claims expressed in this article are solely those of the authors and do not necessarily represent those of their affiliated organizations, or those of the publisher, the editors and the reviewers. Any product that may be evaluated in this article, or claim that may be made by its manufacturer, is not guaranteed or endorsed by the publisher.
